# Postoperative chemoradiotherapy in gastric cancer – a phase I/II dose-finding study of radiotherapy with dose escalation of cisplatin and capecitabine chemotherapy

**DOI:** 10.1038/sj.bjc.6603965

**Published:** 2007-09-11

**Authors:** E P M Jansen, H Boot, R Dubbelman, H Bartelink, A Cats, M Verheij

**Affiliations:** 1 Department of Radiotherapy, The Netherlands Cancer Institute/Antoni van Leeuwenhoek Hospital, Plesmanlaan 121, Amsterdam 1066 CX, The Netherlands; 2 Gastroenterology of the Netherlands Cancer Institute/ Antoni van Leeuwenhoek Hospital, Plesmanlaan 121, Amsterdam 1066 CX, The Netherlands

**Keywords:** gastric cancer, chemoradiotherapy, cisplatin, capecitabine, toxicity, phase I/II study

## Abstract

We hypothesised that gastric cancer outcome could be improved with more effective and intensified postoperative chemoradiotherapy. This phase I/II study was performed to determine the maximal tolerated dose (MTD) and toxicity profile of postoperative radiotherapy with concurrent daily cisplatin and capecitabine. Patients were treated with capecitabine 1000 mg m^−2^ twice a day (b.i.d.) for 2 weeks. Subsequently, patients received capecitabine (250–650 mg m^−2^ orally b.i.d., 5 days week^−1^) and cisplatin (3–6 mg m^−2^ i.v., 5 days week^−1^) according to an alternating dose-escalation schedule. Radiotherapy was given to a total dose of 45 Gy in 25 fractions. Thirty-one patients completed treatment. During chemoradiotherapy, eight patients developed nine items of grade III and one episode of grade IV (mainly haematological) toxicity. The MTD was determined to be cisplatin 5 mg m^−2^ i.v. and capecitabine 650 mg m^−2^ b.i.d. orally. This phase I/II study demonstrated that chemoradiotherapy with daily cisplatin and capecitabine is feasible in postoperative gastric cancer at the defined dose level and is currently being tested in a phase III multicenter study.

Surgical resection remains the cornerstone of curative treatment of gastric cancer (Jansen *et al*, 2005). However, with surgery only, long-term survival is poor, especially in patients with *T*_3–4_ tumours and/or tumour-positive lymph nodes (Wanebo *et al*, 1993; Hundahl *et al*, 2000). This could be, at least in part, accounted for by locoregional relapses that are reported up to 82% (Gunderson and Sosin, 1982). Randomised studies that compared standard D1 lymph node dissections with more extended D2 resections in the Western world, failed to show a significant survival benefit with more extensive surgery (Cuschieri *et al*, 1999; Hartgrink *et al*, 2004). Many studies have been performed to test whether postoperative chemotherapy improves survival. These studies are part of several meta-analyses, which at the best show only a modest survival benefit (Hermans *et al*, 1993; Earle and Maroun, 1999; Mari *et al*, 2000; Gianni *et al*, 2001; Janunger *et al*, 2002). Recently, a substantial increase in survival was found with perioperative chemotherapy in the MAGIC study (Cunningham *et al*, 2006a, 2006b). In this randomised phase III study of 503 patients, three courses of epirubicin, cisplatin and 5-FU (ECF) chemotherapy before surgery and three courses afterwards significantly prolonged progression-free and overall survival (23% for surgery only and 36% with perioperative chemotherapy at 5 years). Earlier, in a trial from the British Stomach Cancer group, no advantage of postoperative radiotherapy only was found (Allum *et al*, 1989). Another strategy to improve survival is postoperative chemoradiotherapy. The Intergroup 0116 study showed in a randomised study of 556 patients that postoperative chemoradiotherapy with 5-FU prolonged 5-year overall survival to 40% as compared to 22% with surgery only (Macdonald *et al*, 2001). This study has been criticised for the fact that 54% of the patients that were included had undergone a D0 lymph node dissection, what can be regarded as suboptimal (Hundahl *et al*, 2002). In this study, which was initiated in the beginning of the 90s of the previous century, chemotherapy was given concurrently only during the first 4 and last 3 days – to a total of 7 days – of radiotherapy, resulting in only a limited interaction between both treatment modalities. We hypothesised that more intensive concurrent treatment with daily cisplatin and capecitabine (which mimics continuous 5-FU infusion) would be more effective (Hoff *et al*, 2001; Evans *et al*, 2002). Therefore, we developed a phase I/II study where conventionally fractionated radiotherapy (45 Gy in 25 fractions, equal to the Intergroup study) was combined with daily oral capecitabine. Cisplatin was added to this regime because of its radiosensitising properties in other malignancies (Schaake-Koning *et al*, 1992; al Sarraf *et al*, 1997; Rose *et al*, 1999; Bernier *et al*, 2004). Both chemotherapeutic drugs were escalated in an alternating fashion.

## MATERIALS AND METHODS

### Patients

Patients with histologically proven adenocarcinoma of stomach or gastro-oesophageal junction in AJCC stage Ib-IV (M0) were eligible for this study (Greene, 2002). Patients with previous malignancies or comorbidity that might compromise delivery of the planned treatment were excluded. Treatment had to be started within 75 days after surgery.

All patients were asked to participate in this study after macroscopically radical gastric surgery was performed. Patients had to be >18 years old, with a WHO performance status of ⩽2. Haematology: haemoglobin ⩾6.5 mmol l^−1^; leucocytes ⩾3.5 × 10^9^ l^−1^; neutrophils ⩾1.5 × 10^9^ l^−1^ and thrombocytes ⩾100 × 10^9^ l^−1^. Renal function: serum creatinine ⩽1.25 ULN and creatinine clearance ⩾60 ml min^−1^ as assessed by 24 h urine collection or calculated by Cockroft and Gault formula. Liver function: total bilirubin ⩽1.5 × ULN; alkaline phosphatase and ASAT/ALAT ⩽3 × ULN. Before treatment, a baseline Tc^99m^-thiatide renogram was performed to evaluate the relative function of the left and right kidney. All patients underwent physical examination, chest X-ray, chest and abdominal CT scans, ECG and evaluation of caloric intake by a dietitian at baseline. A caloric intake of at least 1500 kcal day^−1^ had to be established. During treatment, patients had weekly physical examination, testing of haematological, liver and renal function and determination of weight, caloric intake and toxicity (NCI CTC v3.0). Furthermore, the pathological specimens were reviewed at the Netherlands Cancer Institute.

The study was approved by the Medical Ethical Committee of the Netherlands Cancer Institute, and all patients gave written informed consent.

### Treatment design

The objectives of this phase I/II study were to find the maximal tolerable dose (MTD) of two chemotherapeutic agents, cisplatin and capecitabine, with a fixed radiotherapeutic regimen of 45 Gy and to develop a treatment schedule that could be the experimental arm in a subsequent phase III study. Doses of cisplatin and capecitabine were escalated alternately in dose levels consisting of three patients each. Dose-limiting toxicity (DLT) was defined as any ⩾grade 3 event at any time up to 4 weeks after treatment, except for neutropaenia which was defined as dose-limiting only when being grade IV, with neutropaenic fever, with neutropaenic infection or when it occurred in the first 14 days (capecitabine only) of treatment. When DLT was encountered, an extra group of three patients was treated in the same dose level.

All patients underwent a (partial or total) gastrectomy with preferably at least a D1 lymph node dissection. No routine splenectomy or pancreatic tail resection was done. Whenever possible, jejunostomies were left *in situ* for the entire postsurgical treatment period to facilitate adequate caloric intake.

Because of logistic reasons (waiting time for radiotherapy), all patients started with 2 weeks (days 1–14) of monotherapy with capecitabine 1000 mg m^−2^ twice a day (b.i.d.), after which a non-treatment week (day 15–21) followed. On day 22, radiotherapy started which consisted of 25 fractions of 1.8 Gy to a total dose of 45 Gy in 5 weeks (5 fractions week^−1^). On radiotherapy days, cisplatin (3–6 mg m^−2^ i.v. once daily 1 h before radiotherapy) and capecitabine (250–650 mg m^−2^ orally b.i.d., first dose prior to radiotherapy) were given.

The clinical target volume for radiotherapy consisted of the gastric bed (with stomach remnant when present), anastomoses and the draining lymph nodes, as was described in the Intergroup 0116 study (Macdonald *et al*, 2001). Until the end of 2003, two-field AP-PA techniques (12 patients) were used in treatment, since then multiple field (three-dimensional conformation techniques and/or Intensity Modulated RT (IMRT) techniques (20 patients) were used.

All patients had CT-based dose calculation with construction of dose volume histograms. Dose constraints for critical organs were mean liver dose <30 Gy and for kidneys, at least two-third of one kidney should receive a dose of <40% of the total dose. All patients were treated in a supine position without immobilisation measures on Linacs. Patients were weekly seen by their radiation oncologist and gastrointestinal oncologist. Twice weekly haematology and serum creatinine were checked. All patients were also strictly monitored by a dietitian. Anti-emetics were given on a prophylactic basis, antacid and anti-diarrheic drugs were prescribed when needed.

## RESULTS

Between December 2002 and March 2006 35 patients were entered in this study. Three patients went off study in an early phase of treatment: one because of a cisplatin allergy with skin reaction which was confirmed after rechallenge; one because of patient refusal to take oral capecitabine and one patient had received para-aortic node irradiation because of bladder cancer in another hospital 20 years before that precluded adequate gastric radiotherapy. Thus, 32 patients started treatment and could be evaluated for acute toxicity. Patient characteristics are summarised in Table 1.

All patients finished the 2 weeks of capecitabine only. One patient developed grade III hand-foot syndrome, which was considered to be not dose-limiting, because it initiated at the capecitabine only phase of treatment and the purpose of this study was to evaluate toxicity of chemoradiotherapy. All but one patient completed the chemoradiotherapy part of treatment. A 68-year-old woman deteriorated during the first week of chemoradiotherapy treatment and was admitted on the intensive care unit with neutropaenic fever and severe mucositis of small bowel leading to bacterial translocation, sepsis and ultimately pulmonary failure. This patient was treated in the highest dose level (cisplatin 6 mg m^−2^; capecitabine 650 mg m^−2^ b.i.d.) and although this patient did not finish treatment according to the protocol, the toxicity was considered dose-limiting. Although a dihydropyrimidine dehydrogenase (DPD) deficiency was suspected, DPD enzyme activity measured in peripheral blood mononuclear cells was not decreased. The patient fully recovered with no signs of disease. In five patients, a dose reduction (of capecitabine) was applied because of neutropaenia (2), chest pain (1); hand-foot syndrome (1) and DPD deficiency (1). Grade III/IV toxicity of the remaining 31 patients is summarised in Table 2. Eight patients developed nine items of grade III toxicity, one patient developed grade I toxicity. There was no toxicity related mortality. DLTs were grade III and IV neutropaenia, grade III thrombocytopaenia and grade III nausea. The MTD therefore was cisplatin 5 mg m^−2^ i.v. and capecitabine 650 mg m^−2^ b.i.d. orally. To get a better view on the spectrum of toxicity at this dose level, two extra patients to a total of 8 were treated in this dose level.

**Figure 1:**
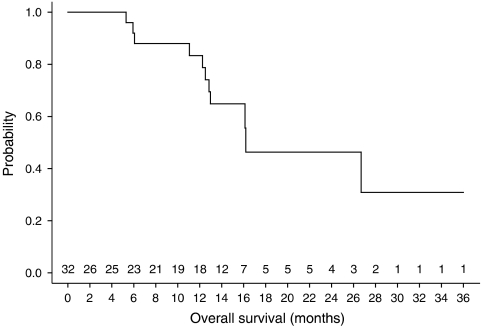
Kaplan–Meier curve of overall survival of all patients that entered the trial.

At time of analysis after a median follow-up of 14.4 (4.8–41.7) months, 20 patients are alive and 11 have died. A Kaplan–Meier plot of overall survival is depicted in Figure 1. One patient died due to local recurrence, seven due to distant metastases and three due to synchronous distant metastases and local recurrence. No patient died due to treatment-related toxicity.

## DISCUSSION

When the Intergroup 0116 trial was initiated at the beginning of the 90s, the concept of concurrent chemoradiotherapy was not as widespread as it is nowadays. Especially, cisplatin-based chemoradiotherapy with daily or weekly administration has proven to be effective in a wide range of malignancies like head and neck, lung and uterine cervix cancer (Schaake-Koning *et al*, 1992; Rose *et al*, 1999; Bernier *et al*, 2004). Furthermore, daily administration of 5-FU analogues has become much easier with the introduction of the oral fluoropyrimidines such as capecitabine. In metastatic colorectal cancer, capecitabine has shown to be at least as effective and to have a favourable side effect profile when compared with intravenous 5-FU (Hoff *et al*, 2001; Cunningham *et al*, 2006b). Capecitabine concurrent with radiotherapy has shown to be feasible and capable of inducing relevant tumour responses in upper GI and rectal cancer (Vaishampayan *et al*, 2002; Rodel *et al*, 2003).

We therefore designed this phase I/II study where a fixed radiotherapy regimen comparable to the Intergroup 0116 trial, was combined with daily cisplatin and capecitabine during weekdays. Capecitabine has been used in gastric cancer in the epirubicin/cisplatin/capecitabine (ECC) regimen with adequate resorption in patients with or without gastric resection (Evans *et al*, 2002).

We demonstrated that postoperative chemoradiotherapy with daily cisplatin and capecitabine during weekdays combined with 45 Gy radiotherapy in 25 fractions is feasible (after 2 weeks of capecitabine monotherapy). The recommended dose for further studies are for cisplatin 5 mg m^−2^ i.v. daily and for capecitabine 650 mg m^−2^ b.i.d. orally. Ninety-seven percent of patients completed the planned treatment. Although we want to emphasise that comparison of our phase I/II toxicity data with large phase III studies is not appropriate, it is stressed that this is in clear contrast to the 64% of patients receiving planned treatment in the Intergroup 0116 trial and 42% of patients in the MAGIC trial. It may reflect the fact that (i) our initial doses of chemotherapy were relatively low, (ii) more conformal radiotherapy techniques were used, (iii) these large phase III trials were multicentre trials and ours is a single institution trial with strict monitoring of the patients, and (iv) underscores the importance of intense supportive care (Macdonald *et al*, 2001; Cunningham *et al*, 2006a). Furthermore, in the MAGIC protocol, chemotherapy was also applied preoperatively, which could diminish tolerability of postoperative treatments. In the Intergroup 0116 study 54% of chemoradiotherapy patients developed ⩾grade III haematological toxicity and in 33% gastrointestinal toxicity, ultimately resulting in 17% of patients withdrawing from the protocol. In the MAGIC study ⩾grade III haematological toxicity consisted mainly of granulocytopaenia (24–28%), lymphocytopaenia (17–20%) and leucopaenia (11–12%). Gastrointestinal toxicity was somewhat lower with about 16% ⩾grade III nausea and 14% vomiting.

In the radiotherapy part of treatment only modifications in clinical target volume delineation have been introduced. In the beginning of the study, two field AP-PA (anterior-posterior) techniques were used like in the Intergroup 0116 study, whereas in the latter part this was replaced by more sophisticated three-dimensional conformal and IMRT techniques. In a dose planning study, we were able to decrease the dose to the (left) kidney while adequately covering the planning target volume (Verheij *et al*, 2005; Jansen *et al*, 2007).

Currently, it is not clear what the optimal strategy to pursue is. Although both the Intergroup 0116 and the MAGIC study have contributed in improving the results of gastric cancer treatment, many questions remain about the optimal treatment. As mentioned before, the Intergroup 0116 study has been criticised for its suboptimal surgery, suggesting that chemoradiotherapy was only balancing this. However, in an observational study from South Korea in nearly 1000 patients who all had a D2 lymph node dissection, it was shown that postoperative chemoradiotherapy could prolong survival and decrease the recurrence rate (Kim *et al*, 2005). Especially questions about the optimal type and sequencing of chemotherapy and the implementation of new radiotherapy and surgical techniques remain. Therefore, we have designed a multicentre phase III study, of which accrual already has begun, in which all patients receive three preoperative courses of ECC, then have gastric surgery followed by another three courses of ECC or chemoradiotherapy (http://www.clinicaltrials.gov/ct/show/NCT00407186). In the experimental arm (chemoradiotherapy) cisplatin and capecitabine dosages will be used that were defined in this phase I/II study. Furthermore, surgery requires at least a D1 resection with at least 15 lymph nodes removed in this study. Above that, quality assurance of surgery (Maruyama index) and radiotherapy will be part of this study.

In conclusion, the combination of modern radiotherapy with daily cisplatin and capecitabine is safe with manageable toxicity in patients who have had curative gastric surgery.

## Figures and Tables

**Table 1:** Patient characteristics (*n*=32)

Sex (M/F)	22/10
Mean age (range; years)	53 (37–73)
	
*Histology*	
Adenocarcinoma	30
Signet cell carcinoma	2
	
*Location of primary tumour*	
Gastro-oesophageal	5
Stomach	27
	
*Surgery*	
Partial gastrectomy	15
Oesophagogastrectomy	6
Total gastrectomy	11
	
*Lymph node dissection*	
D0	13
D1	13
D2	6
	
*pT-stage*	
T1	1
T2	3
T3	24
T4	4
	
*pN-stage*	
N0	3
N1	15
N2	10
N3	4

**Table 2:** Grade III/IV toxicity in relation to chemotherapy dose level during chemoradiotherapy in 31 patients that completed treatment as planned

**Dose level**	**Cisplatin dose (mg m^−2^)**	**Capecitabine dose (mg m^−2^)**	***n***	**Neutropaenia**	**Thrombocytopaenia**	**Nausea**	**Hand-foot syndrome**	**Dysphagia**	**Fatigue**
I	3	250	3						
II	4	250	3						
III	4	350	3						
IV	4	500	3	1			1		
V	5	575	6	1^a^				1	
VI	5	650	8	1		1			
VII	6	650	5		2	1			1

a Grade IV.
